# Comparison of osteogenesis of bovine bone xenografts between true bone ceramics and decalcified bone matrix

**DOI:** 10.1007/s10856-022-06696-x

**Published:** 2022-10-15

**Authors:** Gang Xu, Ruizhou Guo, Liwei Han, Xiaomei Bie, Xiantong Hu, Li Li, Zhonghai Li, Yantao Zhao

**Affiliations:** 1 Department of Orthopaedics, First Affiliated Hospital of Dalian Medical University, Dalian, 116011 PR China; 2 Key Laboratory of Molecular Mechanism for Repair and Remodeling of Orthopaedic Diseases, Liaoning Province, Dalian, 116011 PR China; 3 Institute of Orthopedics, Fourth Medical Center of the General Hospital of CPLA, 100048 Beijing, PR China; 4 Beijing Engineering Research Center of Orthopedics Implants, 100048 Beijing, PR China; 5 State Key Laboratory of Military Stomatology, School of Stomatology, The Fourth Military Medical University, Xi’an, 710032 PR China

## Abstract

Xenograft bone scaffolds have certain advantages such as mechanical strength, osteoinductive properties, sufficient source and safety. This study aimed to compare osteogenesis of the two main bovine bone xenografts namely true bone ceramics (TBC) and decalcified bone matrix (DBM), and TBC or DBM combined with bone morphogenetic protein (BMP)-2 (TBC&BMP-2 and DBM&BMP-2). The characteristics of TBC and DBM were investigated by observing the appearance and scanning electron microscopic images, examining mechanical strength, evaluating cytotoxicity and detecting BMP-2 release after being combined with BMP-2 in vitro. The femoral condyle defect and radial defect models were successively established to evaluate the performance of the proposed scaffolds in repairing cortical and cancellous bone defects. General observation, hematoxylin and eosin (HE) staining, mirco-CT scanning, calcein double labeling, X-ray film observation, three-point bending test in vivo were then performed. It indicated that the repair with xenograft bone scaffolds of 8 weeks were needed and the repair results were better than those of 4 weeks whatever the type of defects. To femoral condyle defect, TBC and TBC&BMP-2 were better than DBM and DBM&BMP-2, and TBC&BMP-2 was better than TBC alone; to radial defect, DBM and DBM&BMP-2 were better than TBC and TBC&BMP-2, and DBM&BMP-2 was better than DBM alone. This study has shown that TBC and DBM xenograft scaffolds can be more suitable for the repair of cancellous bone and cortical bone defects for 8 weeks in rats, respectively. We also have exhibited the use of BMP-2 in combination with DBM or TBC provides the possibility to treat bone defects more effectively. We thus believe that we probably need to select the more suitable scaffold according to bone defect types, and both TBC and DBM are promising xenograft materials for bone tissue engineering and regenerative medicine.

**Figure:**
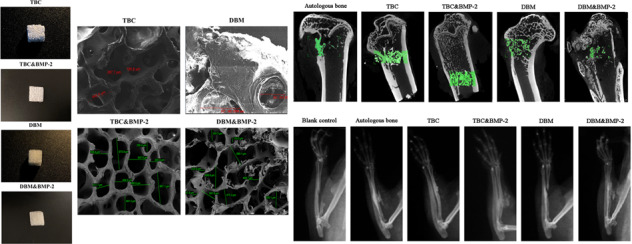
Graphical abstract

Graphical abstract

## Introduction

Bone defects caused by various reasons, such as a high-energy trauma or the bone tumor resection, are common in clinical practice. When the length or diameter of the bone defect reaches to a certain level, it usually cannot heal itself. Autologous bone grafting is the gold standard for bone defect repair due to its excellent bone conduction, osteoinduction and osteogenesis capabilities [1–3]. However, the autologous bone has shortcomings such as the donor pain and local complications such as hematoma, fracture, infection and so on [4], and its clinical application has the limitation. In contrast, allogeneic bone has similar properties without problems such as donor complications, which are naturally favored by researchers. Allografts have long been used as natural substitutes to cover bone defects due to their excellent osteogenesis and bone conduction properties [5], but allogeneic bones also exist defects such as disease transmission, bone nonunion, graft absorption and so on [1, 4]. In order to cope with the above problems, xenogeneic bone seems to be a reliable choice. Compared with autologous bone and allogeneic bone, xenograft bone also has certain mechanical strength and osteoinductive properties, and has the sufficient source and safety. It can rely on physical or chemical means to eliminate or reduce immunogenicity. Common xenograft bones include true bone ceramics (TBC), decalcified bone matrix (DBM), freeze-dried bone (FDB) and so on.

Xenograft TBC is a natural biological scaffold material of calcium phosphate, which is formed from fresh bovine cancellous bone by secondary sintering at high temperature. Its crystallization characteristic is similar to that of artificial hydroxyapatite. The porosity of TBC is high, and the porous structure helps to increase the surface area and facilitate the release of cytokines to the nearby cells [6]. The porous structure also provides space for the growth of new bone and accelerates the growth of osteoblasts. The degradation of TBC releasing calcium and phosphate ions also provides the basis for the formation of new bone. Compared with the synthetic hydroxyapatite, osteoblasts cultured with TBC showed the higher alkaline phosphatase activity [7]. Due to its excellent biocompatibility and bone conduction ability, TBC has the osteoinductive activity after being combined with bone morphogenetic protein (BMP)-2-related peptide [8], rhBMP-2 and Sr [9] and (DSS)6-liposome/CKIP-1 siRNA [10] recently, so as to promote the repair of bone defects better.

Xenograft DBM also is a structure with high porosity, which is conducive to the growth of cells and the diffusion of biological factors [11]. In addition, xenograft DBM also has the excellent biocompatibility, and cytokines buried in the mineralized matrix is more easily exposed to the defect due to the removal of cortical bone during the decalcification process. DBM might also promote osteogenesis through the bone conduction mechanism [12]. The bovine DBM implanted into the radial defect of rabbits have achieved good bone healing without complications [13, 14]. Xenograft DBM could even achieve the same bone defect repair effect as the same as the autologous cortical bone by increasing the mineralized volume of the defect site [15, 16]. However, some studies have also shown that DBM alone was not enough to promote the healing of bone defects [17–19]. At present, the effect of DBM combined with BMP-2 on osteogenesis in vivo is still not clear at all.

Herein, we compared the osteogenic effect of two kinds of xenograft bovine bone scaffolds between TBC and DBM combined with BMP-2 or not in the rat bone defects model to 1) observe the repair result of DBM combined with BMP-2 in vivo and 2) confirm which xenograft or what form is better.

## Materials and methods

### Preparation of the scaffold

#### Preparation of TBC

Preparation of TBC was referred as previously described [8, 20, 21] and modified according to our previous patent [22]. The femoral condyle was partially sawed from the femur, and the soft tissue attached to the bone was peeled off, and then cut into 5 × 5 × 5 mm bone pieces by a cutter and washed with a high pressure water gun. The washed bone pieces were immersed in 0.5 mol/L NaOH solution for 1 h, and ultrasonically washed by the purified water for 3 times, 10 min each time. Then, the bone pieces were immersed in a 3% H_2_O_2_ solution for 30 min and washed as above. The washed bone pieces were boiled in a beaker for 1 h to remove some of the protein and lipids, and the bone pieces were cooled and dried overnight. The dried bone pieces were placed in a muffle furnace for calcination, and the temperature was slowly raised to 770 °C, calcined for 2 h. After the bones were naturally cooled, they were placed in a pulverizer for grinding and powdering. According to our preliminary experiment, the bone powder with a particle size of 270–1000 μm was screened and placed in a ziplock bag. After being sterilized by Co60 (25 kGy), it was stored in a refrigerator at 4 °C for being combined with BMP-2.

#### Preparation of DBM

Preparation of DBM was also referred as previously described [23–25]. The above washed 5 × 5 × 5 mm bone pieces were ultrasonically cleaned in 5% triton according to the bone mass/solvent ratio of 1 g/ml for 30 min, then ultrasonically washed and degreased with methanol/chloroform (1:1). After being degreased, decalcification was carried out by reacting 0.5 mol/ml hydrochloric acid (1:20 g/ml in the shaker at 100 r for 2 h, adding 1:3000 pepsin for ultrasonication for 30 min, and shaking for 8 h. After 30 U/ml α-galactosidase, the mixture was sonicated for 30 min, and shaken for 8 h. 50 U/ml DNase was mixed for 30 min, and shaken for 12 h. In the ultrasonic cleaning process, ice cubes were added to keep the temperature below 37 °C. After completing the enzyme treatment, 75% alcohol was added and shaken at 100 r/min for 15 min. Finally, the sample was lyophilized and made into the bone powder with a particle size of 270–1000 μm according to our preliminary experiment, screened into a ziplock bag, sterilized by irradiation with Co60 (25 kGy), and then stored at 4 °C for further being combined with BMP-2.

### In vitro analysis

#### Mechanical strength testing

The scaffolds were placed on the biomechanical testing machine (MTS-858 Mini Bionix, Minn. America) for fixation, and the maximum compressive load and yield load of the scaffold materials were tested separately. The ambient temperature was 18 °C, air humidity was 50%, and loading speed was 2 mm/min.

#### Scanning electron microscopy (SEM)

According to references [8, 21], MC3T3-E1 cell suspension (2 × 10^5^/ml, 200 μl) was added to the scaffold materials. After incubation at 37 °C for 4 h, the scaffold was covered with cell culture medium containing 10% FBS. The scaffold was taken out from the incubator after 2 days, and fixed with 2.5% glutaraldehyde buffer solution at 4 °C overnight. Then, it was dehydrated through a series of gradients. After drying, the scaffold was sputter-coated with a layer of gold at a thickness of approximately 20 nm. The surface morphology and fine structure of the scaffold materials were observed using a SEM.

#### Cytotoxicity testing

The scaffold materials were weighed and immersed in α-MEM solution at 0.2 g/ml in a 37 °C incubator for 48 h to obtain the extraction solution of scaffolds. The primary cultured rat osteoblasts were replaced with the extraction liquid of the scaffolds. After 7 days, CCK-8 reagent (10 μl) was added to the culture medium for 4 h, and detected at the wavelength of 450 nm. The relative growth rate (RGR) of primary osteoblasts was calculated to evaluate potential cytotoxicity.

#### Scaffolds combined with BMP-2

The powder of the scaffold was weighed, half of which was blended into BMP-2 according to the proportion of 0.5 mg/g, with PBS solution going over the mixed powder. Then, it was continuously blended in the shaking bed for 10 min, after precool for 30 min, the composite scaffold was obtained by lyophilization.

#### Release of BMP-2

The prepared scaffolds were incubated in 200 ml PBS at 37 °C for 24 days. The eluates were collected at 2 h, 12 h and 1, 2, 3, 4, 5, 8, 10, 12, 14, 16, 18 and 14 days after incubation. After removing the supernatant, fresh buffer was supplemented at each time point [20]. The levels of BMP-2 in the supernatant were detected by ELISA.

### In vivo analysis

#### Experimental animals

Goats [26], dogs [27] and rabbits [28] were also often selected as experimental animals to achieve the model of femoral condyle defect. Compared with these experimental animals, rats were cheap, convenient for surgical operation and subsequent sampling, and had strong anti-infection ability, and were less likely to suffer from surgical site infection and death. Therefore, rats were selected as the experimental animals in this study.

Seventy-two SD rats (300–350 g, male) were provided and raised by the Fourth Medical Center of PLA General Hospital. The environment was free of pathogens, the ambient temperature was 22 °C, 12 h light /12 h dark cycle was guaranteed every day, the indoor humidity was 50–55%, and adaptive feeding was carried out for one week before the experiment. The animal experiments were approved by the Animal Ethics Committee of the Fourth Medical Center of PLA General Hospital, and were conducted in accordance with NIH guidelines (or for non-U.S. residents similar national regulations) for the care and use of laboratory animals (NIH Publication #85-23 Rev. 1985).

#### Surgical procedures

Seventy-two SD rats were randomly divided into 6 groups (*n* = 12 per group), namely: blank control, autologous bone, TBC, TBC&BMP-2, DBM and DBM&BMP-2. Half of the rats each group were selected to construct a femoral condyle defect. Briefly, in the left knee joint area, skin preparation, disinfection and towel placement were performed routinely. The lateral longitudinal incision of the patella was made, with a length of about 1.5 cm. Skin, muscle and joint capsule were cut layer by layer, and a cylindrical defect (diameter = 3 mm, depth = 5 mm) was drilled into the anterior lateral wall of the lateral condyle of the femur using a medium-speed grinding drill. The defect was formed by rotation via a hemostatic forceps, and the deep residual bone mass was removed with small curette.

The remaining rats in the groups were used to construct a radial defect model. The most commonly selected site for the study of segmental long bone defects was the middle humeral shaft [29]. This part was not required to be fixed and the tibia could be connected to the ulna by the interosseous membrane and fixed by the ulna. Briefly, in the left forelimb area, skin preparation, disinfection and towel placement were performed routinely. The median incision of the forelimb was made, with a length of about 1.0 cm. Skin and muscle were cut and separated, and a 4-mm bone segment defect (about 1.0 cm away from the distal radius) was formed by bone scissors and a tape.

These two kinds of defects were filled with no scaffolds, autologous bone, TBC, TBC&BMP-2, DBM and DBM&BMP-2. As for evaluation methods, it mainly relied on general observation, HE staining, Mirco-CT and X-ray scanning and the relative parameters. The grading method of Hitchcock [30] was used to evaluate the degree of tendon adhesion in each group. The grading scores were calculated after 8 weeks of stent implantation.

#### Micro-CT observation

At the eighth week postoperation, the femoral condyle (about 6 mm) was taken out from the femoral condyle to the distal femoral shaft by animal bone scissors. Micro-CT (Inveon MM CT) scanning was conducted, and the obtained data were analyzed with Inveon Acquisition Workplace. The selected region of interest was 2 mm in diameter and 5–8 mm in depth.

#### X-ray film observation

At the eighth week postoperation, the left forelimb of the rat was photographed by X-ray. The formation of new bone, bone healing, shaping and scaffold in the defect area of the tibia were observed, and scored according to the Lane–Sandhu scoring system as follows: 0% no callus tissue, fracture line is clear; 2–25% callus tissue, fracture line is still clearly visible; 5–50% callus tissue, fracture line is blurred; 8–75% callus tissue, fracture line is barely visible; and 10–100% callus tissue, no remaining fracture line is visible. The scores given by two independent observers who were blinded to the experimental design were averaged to obtain the final score [31].

#### Histological analysis

##### Hematoxylin-eosin (HE) staining

At the fourth and eighth week postoperation, the rats were sacrificed. The implanted scaffolds and surrounding bones were removed and immersed in 10% neutral buffered formalin fixative solution. After fixation, dehydration and decalcification, the cut surface was placed at the bottom for paraffin embedding, sliced at a thickness of 5 μm, and then stained with hematoxylin-eosin (HE) for observation. The histological scoring criteria proposed by Nilsson were used [32]. Semi-quantitative analysis was performed by two investigators who were blinded to the experimental design.

##### Calcein staining

Three rats in each group were subcutaneously injected with calcein at a dose of 9 mg/kg, 5 and 2 days before the eighth week postoperation. The specimens were collected and fixed in 10% neutral buffered formalin fixative. After 7 days, the specimens were taken out and immersed in gradient alcohol for stepwise dehydration, and finally embedding with polymethyl methacrylate. All samples were cut into 10–20 μm sections for histologic examination.

#### Biomechanical detection

The distal radius of each rat was assessed by three-point bending tests using a material testing machine (Tophung, Co. Ltd, Suzhou, China). The maximum load was 15 kg, span of both ends was 30 mm, and loading speed was 2 mm/min. Peak load and maximum deflection were recorded by the computer.

### Statistical analysis

GraphPad Prism 7.00 software was used for statistical analysis and graph plotting. The measurement data were expressed as mean ± standard deviation. Statistical difference between groups was compared by one-way ANOVA. The graded data was counted. *P* < 0.05 was considered statistically significant.

## Results

### Characterization of TBC, DBM, TBC&BMP-2 and DBM&BMP-2

**Fig. 1:**
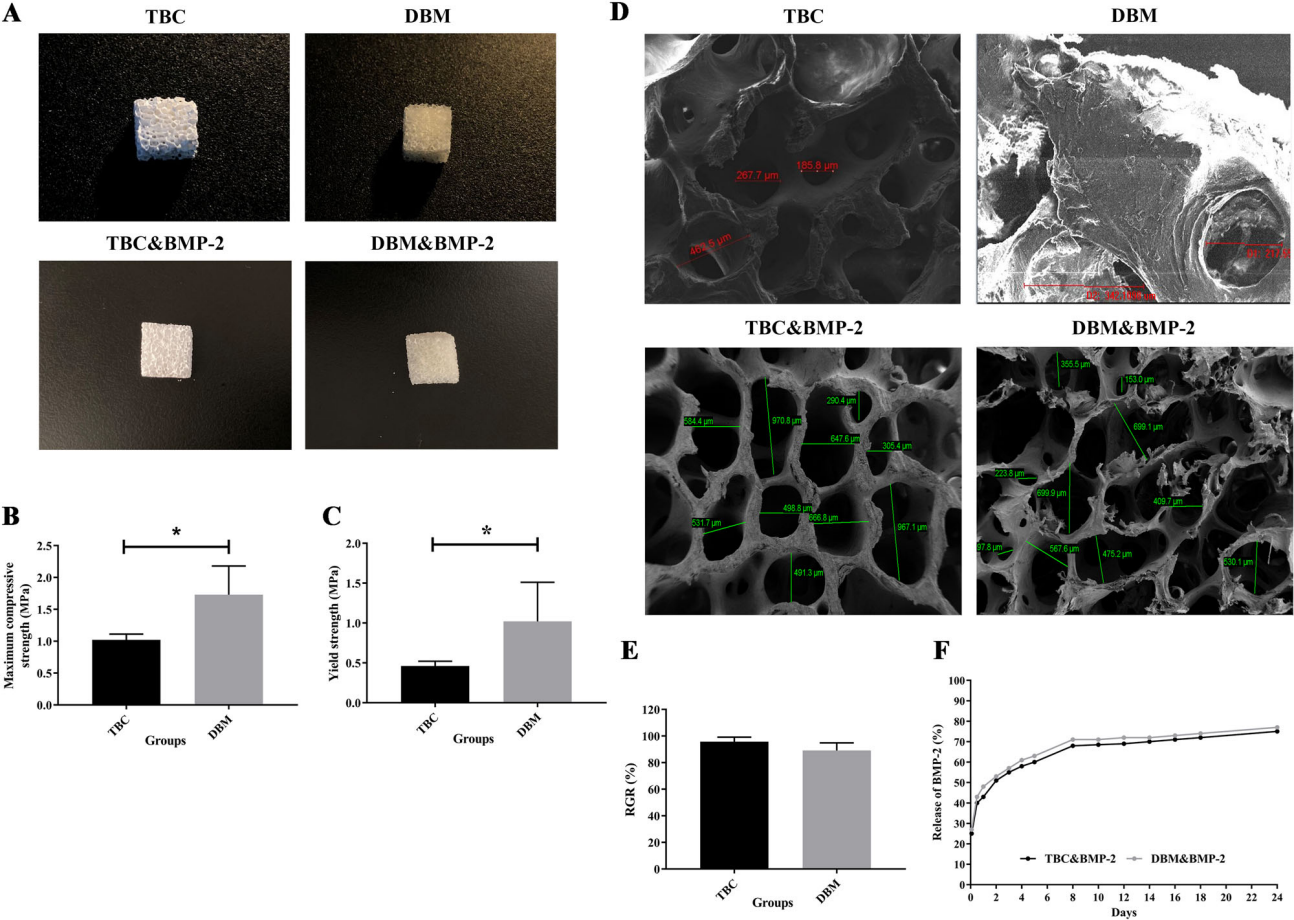
Characterization of TBC, DBM, TBC&BMP-2 and DBM&BMP-2. **A** The photographs of TBC, DBM, TBC&BMP-2 and DBM&BMP-2; **B** The maximum compressive, *n* = 5; **C** The yield strength, *n* = 5; **D** SEM images of TBC, DBM, TBC&BMP-2 and DBM&BMP-2, 100×; **E** The relative cells growth rate (RGR), *n* = 5; **F** The release of BMP-2, *n* = 3. ^*^P < 0.05

The photographs of TBC, DBM, TBC&BMP-2 and DBM&BMP-2, as shown in Fig. 1A. Both the four kinds of xenografts were white, the internal structures of which were loose and porous. Both TBC and TBC&BMP-2 scaffolds were brittler and easier to be crushed, while the DBM and DBM&BMP-2 scaffolds were tougher. The maximum compressive strength of TBC was 1.022 ± 0.09 MPa, which of DBM was 1.73 ± 0.45 MPa, and the comparison was statistically significant (*P* < 0.05) (Fig. 1B). The yield strength of TBC was 0.46 ± 0.06 MPa, which of DBM was 1.02 ± 0.49 MPa and the comparison also was statistically significant (*P* < 0.05) (Fig. 1C).

In addition, SEM images of TBC, DBM, TBC&BMP-2 and DBM&BMP-2, as shown in Fig. 1D. The surface of TBC scaffold was rough, with loose porous structure and the pore size between 185–462 μm, while on the surface of DBM, cracks and raised particles could be seen locally, and the pore diameter was between 217–342 μm. The pore size of TBC&BMP-2 was between 290–971 μm, and the pore diameter of DBM&BMP-2 was between 97–700 μm (Fig. 1D).

The result of cytotoxicity testing by CCK-8 exhibited that RGRs of TBC and BDM were 95.74 ± 3.43% and 89.12 ± 5.68%, and there was no statistical difference (*P* > 0.05) (Fig. 1E). Moreover, according to the cytotoxicity grade, both the RGRs were above 80%, belong to the I level, which could be regarded as no cytotoxicity. Further, we examined the release kinetics of BMP-2 from TBC and DBM, cumulative release amounts of which were as shown in Fig. 1F. During the first 24 h (2, 12, 24), both release rates were rapid, and DBM even more rapid, reached to about 50%. From days 2 to 8, both the release rates were slowed down, added up to nearly 70%. Then, BMP-2 of DBM was gradually released and the release rate decreased along with the time, finally up to around 77% from days 8 to 24, which was a little higher than that of TBC at the different time points (Fig. 1F).

### The repair of femoral condyle defect

The degrees of adhesion at eight weeks, as shown in Table 1. Implanted scaffolds could clearly be observed in both TBC and TBC&BMP-2 groups, and scaffold materials and surrounding bone tissue of which were compactly connected, defect areas of which were hard, the hyperblastosis could be seen in defect areas of TBC&BMP-2. The color of implanted scaffolds was more transparent in DBM and DBM&BMP-2 groups, and there were cracks between scaffold materials and surrounding bone tissue, defect areas of which were more strong but pliable in the textures, fewer hyperplasia tissues could be observed.

**Table 1:** Hitchcock grades count of femoral condyle repair

Grades	Blank control	Autologous bone	TBC	TBC&BMP-2	DBM	DBM&BMP-2
−	6	0	0	0	0	0
+	0	0	0	0	2	3
++	0	1	1	1	4	3
+++	0	5	5	5	0	0

**Fig. 2:**
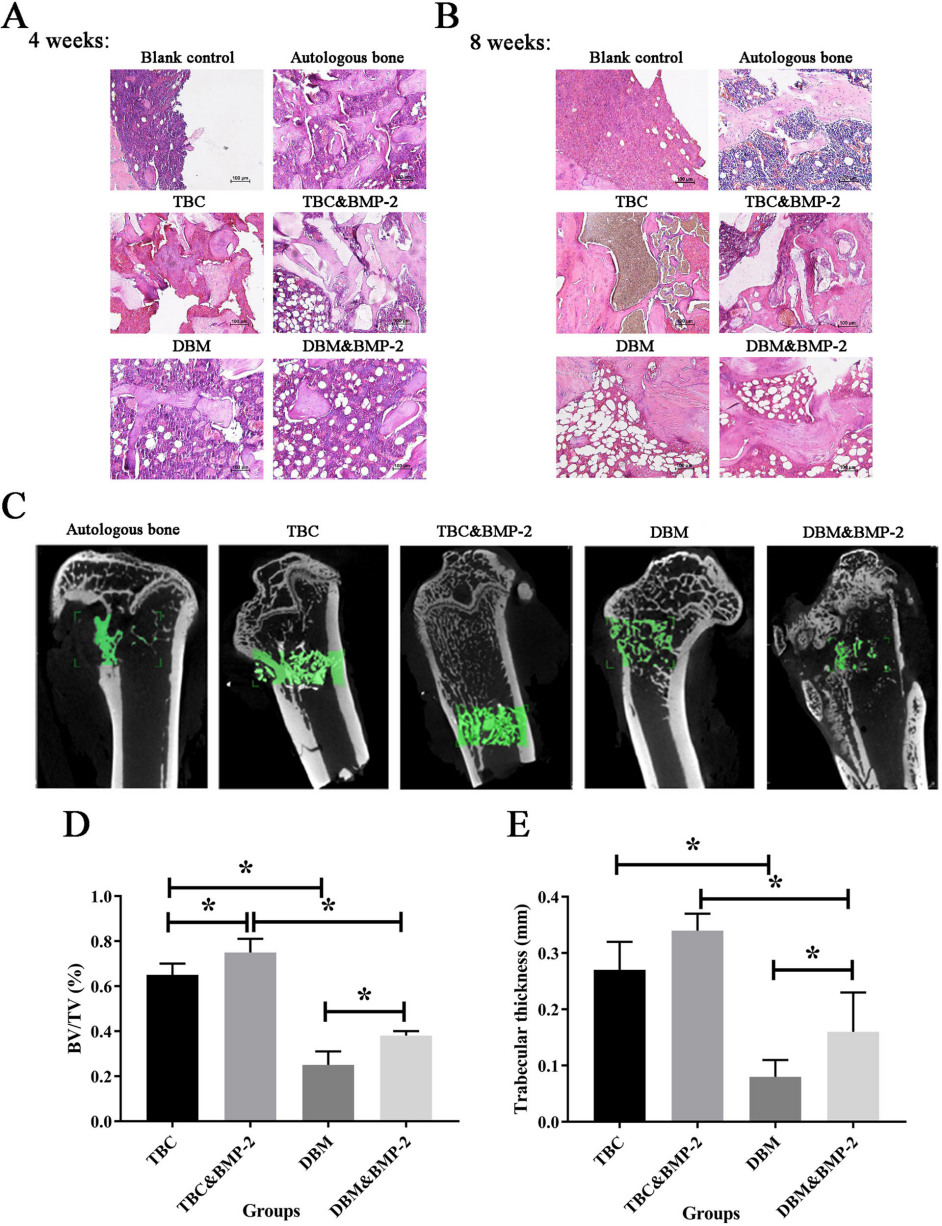
The repair of femoral condyle defect. **A** HE staining of 4 weeks; **B** HE staining of 8 weeks; **C** Micro-CT scanning of 8 weeks, *n* = 6; **D** The bone mass/total volume (BV/TV) scores, *n* = 6; **E** The bone trabecular thickness (TT), *n* = 6. *P < 0.05

At four weeks post-surgery, there was no resorption of the scaffold material in the TBC group, and a small amount of bone callus was observed around the scaffold with the osseous attachment, but no bone marrow formation could be seen. The scaffold material of the TBC&BMP-2 group also was not degraded, there were a large number of formed bone calluses around the scaffold materials with the osseous connection, and the bone marrow was also observed. In the DBM group, there were no formed connection and callus, but the bone marrow area of which was more than 1/2 of the defect area, and there was no significant change after the combination with BMP-2 (Fig. 2A). In accord with the above results, the score of TBC&BMP-2 was higher than that of TBC, DBM or DBM&BMP-2, and there was on obvious enhancement between DBM and DBM&BMP-2 (Table 2).

**Table 2:** Histological scoring of femoral condyle repair

Weeks	Blank control	Autologous bone	TBC	TBC & BMP-2	DBM	DBM & BMP-2
4	1.0 ± 0.0	8.0 ± 0.2	4.0 ± 0.7	6.0 ± 0.7	3.2 ± 0.4	3.4 ± 0.5
8	1.2 ± 0.4	9.0 ± 0.3	5.8 ± 0.4	6.0 ± 0.1	3.6 ± 0.5	4.6 ± 0.4

At eight weeks post-surgery, A large number of bone callus formed around the TBC undegraded scaffold and the osseous connection could be observed, but no bone marrow formation was observed. And no significant change was observed after the combined with BMP-2. In DBM group, a large number of undegraded scaffold materials were observed, but no connection was formed, and the bone marrow area exceeded 1/2 of the defect area. Bone connection was formed in DBM&BMP-2 group, a large amount of callus was formed, and the bone marrow area was more than 1/2 of the defect area (Fig. 2B). The scores of TBC and TBC&BMP-2 were higher than those of DBM and DBM&BMP-2, and these two scaffolds showed no significant improvement after combined with BMP-2 (*P* > 0.05) (Table 2).

In addition, Micro-CT scanning results of the groups at eight weeks post-surgery, as shown in Fig. 2C. It revealed that the Bone Volume/Total Volume (BV/TV) score of TBC&BMP-2 group was the highest (0.75), followed by TBC group (0.65), and the difference between the two groups was statistically significant (*P* < 0.05). The bone mass/total volume (BV/TV) scores of DBM and DBM&BMP-2 were 0.25 and 0.38, and the difference was statistically significant (*P* < 0.05) (Fig. 2D). In line with the results, the bone trabecular thickness (TT) was highest in the TBC&BMP-2 group (0.34 ± 0.03 mm), followed by the TBC group (0.27 ± 0.05), the difference was not statistically significant (*P* > 0.05), the difference between the DBM and DBM&BMP-2 was statistically significant (*P* < 0.05) (Fig. 2E).

Taken together, it appeared that TBC and TBC&BMP-2 were better than DBM and DBM&BMP-2, and DBM&BMP-2 was better than DBM alone, to the repair of femoral condyle defect.

### The repair of radial defect

The rats were generally in good condition after the operation, and there were no abnormalities in diet, water intake, urine and feces. At two weeks after surgery, no oozing fluid, swelling and nonunion occurred around the incision of all rats. However, it was found that all rats appeared some degree of activity restriction. In TBC group, the filled scaffold material was white, hard and closely connected with the boundary of normal bone tissue. No fracture or swelling occurred in the limbs of all rats. There was a small amount of proliferative tissue around the bone defect in TBC&BMP-2 group, the scaffold material of which was fixed in white, with a relatively hard texture and also closely connected to the boundary of normal bone tissue. DBM group could reach the broken end of the bone defect, and the scaffold material was filled in, which was translucent. In DBM&BMP-2 group, the bone defect area was a thin slit, and the two sides of the slit were filled with scaffolds. The texture was soft and dense, and the color was slightly darker than the normal bone tissue. There was a small amount of tissue hyperplasia at the distal end of the bone defect. Adhesion Hitchcock scores of all the groups, as shown in Table 3.

**Table 3:** Hitchcock grades count of radial repair

Grades	Blank control	Autologous bone	TBC	TBC&BMP-2	DBM	DBM&BMP-2
−	6	0	0	0	0	0
+	0	0	0	0	0	3
++	0	0	1	0	2	0
+++	0	6	5	6	4	3

**Fig. 3:**
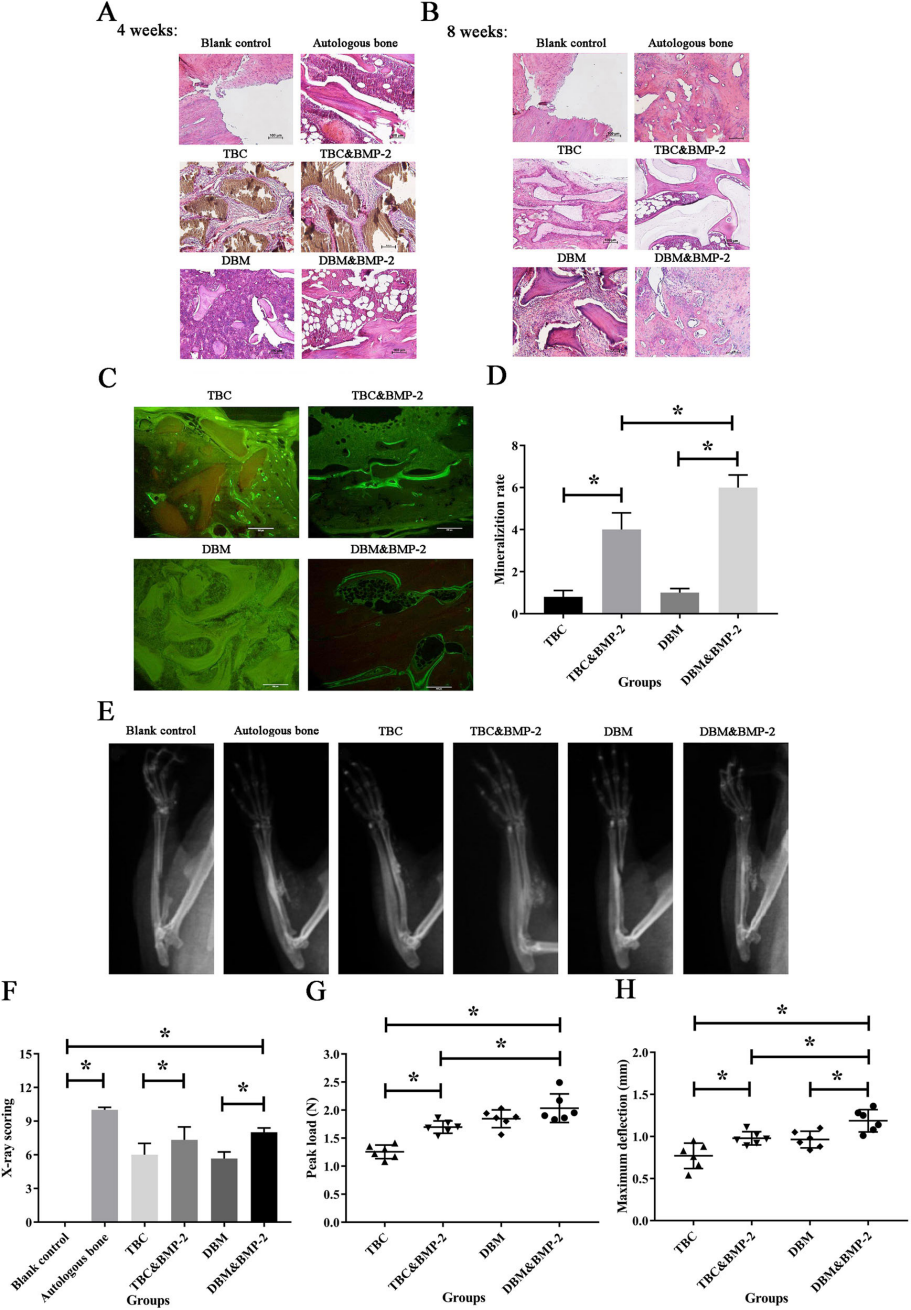
The repair of radial defect. **A** HE staining of 4 weeks; **B** HE staining of 8 weeks; **C** Calcein staining of 8 weeks, *n* = 3; **D** The mineralization and deposition rate, *n* = 6; **E** The X-ray films of 8 weeks, *n* = 6; **F** The X-ray scoring, *n* = 6; **G** The peak load, *n* = 6; **H** The maximum deflection, *n* = 6. *P < 0.05

At the fourth week, a large amount of residual scaffold material was observed in the TBC group, and the defect area showed fibrous connection without callus and bone marrow formation. The defect area in the TBC&BMP-2 group was osseous junction, with a small amount of callus formation, but without bone marrow. The defect area of DBM group was fibrous connection, and a small amount of bone callus was formed. In DBM&BMP-2 group, there was a small amount of residual scaffold material, the defect area showed fibrous connection and a small amount of formed callus, and the bone marrow area of the defect area was more than 1/2 (Fig. 3A). TBC group had the lowest score (0.8 ± 0.4). Autogenous bone group had the highest score (6.0 ± 0.1), followed by DBM&BMP-2 group (4.0 ± 0.1). The early scores of HE staining indicated that the scores of the two scaffolds were improved to different degrees after the combination with BMP-2, and there was a statistical difference between TBC&BMP-2 and DBM&BMP-2 (*P* < 0.05) (Table 4).

**Table 4:** Histological scoring of radial repair

Weeks	Blank control	Autologous bone	TBC	TBC&BMP-2	DBM	DBM&BMP-2
4	0.0 ± 0.0	6.0 ± 0.1	0.8 ± 0.4	3.4 ± 0.5	2.0 ± 0.1	4.0 ± 0.1
8	0.0 ± 0.0	6.0 ± 0.2	1.2 ± 0.4	4.5 ± 0.7	2.4 ± 0.5	6.0 ± 0.2

At the eighth week, fibrous connections were observed around the undegraded material in TBC group, and callus formation was not observed. After TBC was combined with BMP-2, the callus number did not change significantly, but bone marrow began to form. In DBM group, there were fibrous connections and no callus formation. DBM&BMP-2 group presented osseous connection and a large amount of formed callus, the two ends of the defect gradually closed, and the bone marrow area was more than 1/2 of the defect area (Fig. 3B). The scores of DBM&BMP-2 and autologous bone group were the highest (6.0 ± 0.2), followed by TBC&BMP-2 group (4.5 ± 0.7), TBC group had the lowest score of 1.2 ± 0.4 (Table 4).

At the eighth week, no obvious signs of “double rail sign” were found in the scaffold that were not combined with BMP-2, and both the scaffolds presented double rail sign after the combination with BMP-2 (Fig. 3C). Among them, the mineralization rate of DBM&BMP-2 group was the highest, and the difference was statistically significant (*P* < 0.05) (Fig. 3D).

X-ray films of all the groups were taken on eight weeks after the operation. In TBC group, a large number of high-density shadows mixed with scaffold materials and new bone were filled in the entire defect area, with discontinuous bone cortex, visible fracture line and intermedullations, the X-ray score of which was 6.00 ± 1.02. In TBC&BMP-2 group, the scaffold had filled in the most defect area, the fracture line nearly disappeared, and the medullary cavity was formed, the X-ray score of which was 7.33 ± 1.15. There was a significant difference between the two groups (*P* < 0.05). The new bone in DBM group accounted for 50% of the defect area, the fracture line was clearly seen, and no bone shaping was observed, the X-ray score of which was 5.67 ± 0.58. Above 75% of DBM&BMP-2 group was filled with the new bone, the fracture line was still present, and no bone shaping was observed, the X-ray score of which was 8.00 ± 0.39, and there was a significant difference between the two groups (*P* < 0.05) (Fig. 3E, F).

Similarly, the peak load of TBC was smaller than that of TBC&BMP-2, and the latter was smaller than that of DBM&BMP-2, both the comparisons were statistically significant (*P* < 0.05) (Fig. 3G). The maximum deflection of TBC also was smaller than that of TBC&BMP-2, and the latter also was smaller than that of DBM&BMP-2, both the comparisons were also statistically significant (*P* < 0.05) (Fig. 3H).

Taken together, the results indicated that DBM and DBM&BMP-2 were better than TBC and TBC&BMP-2, and DBM&BMP-2 was better than DBM alone, to the repair of radial defect.

## Discussion

Bone graft surgery continues to play a key role in the practice of orthopedic surgery, having a significant clinical and financial impact on our health care system. Autologous bone remains the unmatched gold standard for clinical outcomes, but allogeneic bone and bone graft substitutes require further consideration given their limited supply and increased patient morbidity. Xenografts will continue to be an attractive alternative because they are available in large quantities at lower cost from biologically controlled healthy donors [33]. Nowadays, xenograft-derived bone products are still not FDA approved for use in any orthopedic surgery application, bovine-derived bone has been reported in many clinical results with unfavorable results [33], basic and further researches are essential to investigate and explain the reasons. In this study, we compared osteogenic effects of TBC and DBM on femoral condyle and radial defects. The results indicated that the repair with xenograft bone scaffolds of 8 weeks were needed and the repair results were better than those of 4 weeks whatever the type of defects, which was in line with previous studies [19, 34].

To femoral condyle defect, TBC and TBC&BMP-2 were better than DBM and DBM&BMP-2, and TBC&BMP-2 was better than TBC alone, which was consistent with our previous report using a rabbit model [9]. TBC&BMP-2 were probably more suitable for the repair of cancellous bone due to its rough surface with the loose porous structure and the nice bone conductivity, which was supported by the previous research [7, 35]. Besides, when the scaffold materials were not combined with BMP-2, the osteogenesis of TBC was better, indicating that bone conduction performance played a relatively important role in this model.

However, DBM’s own osteoinductive performance could still play a certain role in promoting osteogenesis. Compared with DBM, the weakness of TBC might lie in the relatively poor degradation performance. At the eighth week, there were still many materials left, which was not conducive to bone remodeling and shaping. The more effective degradation of TBC should be the research emphasis in the future.

To radial defect, DBM and DBM&BMP-2 were better than TBC and TBC&BMP-2, and DBM&BMP-2 was better than DBM alone, which was similar with the previous study that BMP-2-loaded collagen was capable of a critical-size-defect reconstruction in rats instead of demineralized bone matrix [19]. DBM&BMP-2 were probably more suitable for the repair of cortical bone due to its excellent biocompatibility, osteoinduction and mechanical capacity.

In addition, it was also reported that DBM had obvious advantages over chitosan and polymethyl methacrylate in the study of rat tibia bone defects [36]. However, there were few studies on the use of DBM&BMP-2 or TBC alone in this model. Polyamino acid [37] or polylysine [38] was usually used to modify hydroxyapatite, or rhBMP-2 [39] was used for the implantation of tibia defect. It also indicated that in this bone defect model, a scaffold material only with the bone conduction property may not achieve a good osteogenesis effect. According to the results of X-ray observation of this study, although the score of DBM was not so high as expected, this may be related to the poor uniformity of DBM after decalcification [40]. Combined with the results of HE staining and calcein double labeling, it could be considered that the DBM/BMP-2 scaffold with the good osteoinductive activity could achieve better radial osteogenic effects.

As to the selection of critical bone defect size, for long tubular bone, due to the difference in anatomical development, the length of a certain size was unreliable. The critical bone defect size of this model needs to be determined by the diameter of the long bone. The length of the bone defect was generally considered to be a limit bone defect at 1.5–2.0 times the diameter of the long bone backbone [41]. In the preliminary experiment, we measured that the diameter of the tibia shaft of the 300–350 g rat was 2.0 ± 0.5 mm, so we believed that 4 mm had met the limit bone defect size of the rat tibia, which was consistent with the reference [19]. In the preparation of the bone defect model, in order to eliminate the influence of periosteum on osteogenesis, we also removed the periosteum in this area while observing the bone segment to avoid false positive results caused by periosteum.

BMP-2 plays an important role in bone regeneration to TBC or DBM. Bone morphogenetic proteins (BMPs) are members of the transforming growth factor-β superfamily with the strong bone induction activity [8, 9, 19, 20]. Accordingly, recombinant human BMPs have been developed as osteoinductive reagents to repair bone defects clinically [42]. The application of BMPs has recently emerged as an effective treatment in bone reconstruction surgery [6]. Among BMPs, BMP-2 exhibits the strongest activity of inducing bone-regeneration and has been used clinically [6, 8]. In this regard, a novel scaffold containing TBC combined with sustained delivery of BMP-2 related peptide (P28) have been reported, it could promote proliferation and osteogenic differentiation of MC3T3-E1 cells in vitro and new bone tissue generation in vivo [8]. Another surface mineralization-modified TBC scaffold combined with BMP-2-related peptide (P24) could enhance the osteoblastic differentiation of cells and promote significant bone defect repair compared with TBC scaffolds containing no BMP-2 [20]. With BMP-2-loaded collagen as the control, demineralized bone matrix was not capable of a 4 mm femur defect reconstruction, however, BMP-2 was able to achieve it [19]. Moreover, high BMP-2 doses could prevent bone overgrowth and ectopic bone formation in a negative feedback loop through the Wnt signaling pathway to guarantee the safety [43].

One of the limitations of our experimental study is that we were failed to ensure that scaffolds had the same surface characteristics between TBC and TBC&BMP-2, or between DBM and DBM&BMP-2. Taking the function of BMP-2 into consideration, it may play more roles in this study.

## Conclusion

In summary, this study has shown that TBC and DBM xenograft scaffolds can be more suitable for the repair of cancellous bone and cortical bone defects for 8 weeks in rats, respectively. We also have exhibited the use of BMP-2 in combination with DBM or TBC provides the possibility to treat bone defects more effectively. We thus believe that we probably need to select the more suitable scaffold according to bone defect types, and both TBC and DBM are promising xenograft materials for bone tissue engineering and regenerative medicine.
